# Retrospective evaluation of the contribution of radiotherapy to survival in breast cancer treatment with propensity score based on stage and subgroup

**DOI:** 10.1186/s13014-024-02474-x

**Published:** 2024-06-26

**Authors:** Rusen Cosar, Necdet Sut, Sule Parlar, Yıldıray Ozguven, Dilek Nurlu, Ebru Tastekin, Sena Batu, Eylül Şenödeyici, Talar Ozler, Melisa Dedeli, Gökay Yıldız, Sekip Kavukcu, Mert Chousein, Zeynep Alas, Sernaz Topaloglu

**Affiliations:** 1 Faculty of Medicine, Department of Radiation Oncology, Trakya University, Edirne, Turkey; 2 Faculty of Medicine, Department of Biostatistics, Trakya University, Edirne, Turkey; 3 Faculty of Medicine, Department of Medical Physics, Trakya University, Edirne, Turkey; 4 Faculty of Medicine, Department of Pathology, Trakya University, Edirne, Turkey; 5 Trakya University Faculty of Medicine, Edirne, Turkey; 6 Faculty of Life Sciences-Molecular and Cellular Biology, Strasbourg University, Strasbourg, France; 7 Faculty of Medicine, Department of Medical Oncology, Trakya University, Edirne, Turkey

**Keywords:** Breast cancer, Luminal B breast cancer, Molecular subtypes radiation therapy, Propensity score, Regional nodal irradiation in breast cancer

## Abstract

**Background:**

Breast cancer has been a disease in which treatment strategy has changed over time under the influence of different hypotheses and evidence for more than a century. We analyzed the contribution of radiotherapy to disease-free survival and overall survival by classifying according to stage, 1–3 lymph node involvement, and molecular subgroups.

**Methods:**

Following the approval of the Institutional Review Board, records of patients with breast cancer who were admitted to University School of Medicine Departments of Radiation Oncology and Medical Oncology between July 1999 and December 2020 were reviewed. Using data propensity score matching was performed between the groups that did and did not receive radiotherapy using an optimal matching algorithm (optimum, 1:1). Disease-free survival and overall survival after propensity score matching were calculated using the Kaplan-Meier method. Univariate and multivariate Cox regression analysis was used to estimate hazard ratios.

**Results:**

In the radiotherapy and non-radiotherapy groups, disease-free survival was 257.42 ± 5.46 (246.72- 268.13), 208,96 ± 8,15 (192,97–224,94) months respectively, (*p = < 0.001)*, overall survival was 272,46 ± 8,68 (255,43–289,49), 219,05 ± 7,32 (204,70–233,41) months respectively (*p* = .002). We compared the 19 N1 patient groups who received radiotherapy with the 19 patients who did not receive radiotherapy and calculated the disease-free survival times was 202,21 ± 10,50 (181,62–222,79) and 148,82 ± 24,91 (99,99–197,65) months respectively (*p* = .011) and overall survival times was 200,85 ± 12,79 (175,77–225,92) and 166,90 ± 20,39 (126,93–206,82) months respectively (*p* = .055). We examined disease-free survival and overall survival times in both groups according to Luminal A, Luminal B, TNBC, and HER2-enriched subgroups. In the Luminal B subgroup, the disease-free survival duration in the groups receiving radiotherapy and not receiving radiotherapy was 264.83 ± 4.95 (255.13-274.54) and 187.09 ± 11.06 (165.41-208.78) months (*p* < .001), and overall survival times were 252.29 ± 10.54 (231.62-272.97) and 197.74 ± 9.72 (178.69–216.80) months (*p* = .001) respectively.

**Conclusions:**

Thanks to studies proving that RT increases long-term survival rates in breast cancer as a result of reducing locoregional recurrence and systemic metastasis rates, it has been understood that the spectrum hypothesis is the hypothesis that most accurately describes breast cancer to date. We found that patients with Luminal B invasive breast cancer benefited significantly more from RT compared to other subgroups.

**Supplementary Information:**

The online version contains supplementary material available at 10.1186/s13014-024-02474-x.

## Introduction

Breast cancer has been a disease in which treatment strategy has changed over time under the influence of different hypotheses and evidence for more than a century [1]. First, the surgical treatment of breast cancer has evolved from aggressive surgical interventions to much more comfortable minor surgeries with the contribution of post-operative radiotherapy (RT) [2, 3]. With the introduction of systemic treatments, death rates due to metastasis decreased and survival rates increased [4]. It has been proven by both animal studies and randomized multicenter studies that breast cancer is a local, nodal and systemic disease [5–8]. A 30-year-long evaluation of the Danish Breast Cancer Cooperative Group (DBCG) 82bc study shows that post-mastectomy radiotherapy (PMRT) enormously improves loco-regional tumor control and subsequent survival [9]. Hellman has been the biggest supporter of the spectrum hypothesis, proving that local-nodal disease without RT predisposes metastasis development [10, 11].

Despite the numerous changes and advancements in breast cancer treatment, the rate of axillary lymph node involvement is still the most important prognostic indicator for patients with breast cancer [3, 12, 13]. Since the involvement of lymph nodes in breast cancer is of biological importance, the type of surgery the patient has undergone, the number of axillary lymph nodes involved, and radiotherapy remain the primary determinants in treatments decisions [14–16].

There are differences even between guidelines in the current indication for RT in invasive breast cancer. The American Society of Clinical Oncology, the American Society of Radiation Oncology, and the Society of Surgical Oncology [17] recommend that RT be applied to all patients with invasive breast cancer treated with breast-conserving surgery, except for those over 70 years of age and estrogen receptor (ER) positive, grade I. While the National Institute for Health and Care Excellence (NICE) [18] defines the group in which RT should be applied, it recommends adjuvant RT to all patients with positive axillary lymph node involvement after mastectomy. However, St. Gallen, at the International Expert Consensus Conference on the Primary Treatment of Early-Stage Breast Cancer, recommends that RT should be considered for patients with invasive breast cancer who have T1-2 tumors and one to three positive lymph node involvement after mastectomy [16–19]. We understand that RT in breast cancer is a treatment that is difficult to give up when lymph node involvement is present despite advanced chemotherapy options. Based on this, we focused on the group of patients who did not receive RT in our invasive breast cancer series, which has long and thorough follow-up data. We tried to measure the effect of RT on disease-free survival (DFS) and overall survival (OS) by creating a similar group with our RT-treated patient group of 189 breast cancer patients who did not receive RT. Reasons for not applying RT for 189 patients who did not receive RT were the refusal of RT by the patient and surpassing the 6-month adjuvant treatment period, where RT is not recommended. To measure the effect of RT, we included 189 patients with invasive breast cancer who did not receive RT and 189 patients who received RT. We analyzed the contribution of RT to DFS and OS by classifying according to stage, 1–3 lymph node involvement, and molecular subgroups.

## Material and method

Following the approval of the Institutional Review Board, records of patients with breast cancer who were admitted to Trakya University School of Medicine Departments of Radiation Oncology and Medical Oncology between July 1999 and December 2020 were reviewed. The Human Research Ethical Committee of Trakya University Medical Faculty Hospital approved (TUTF-GOBAEK 2023/195) using these patients’ information for the study. In order to use the relevant information, informed consent forms were obtained from the patients or relatives of the deceased patients from our local ethics committee in accordance with the Declaration of Helsinki [20].

This study was modeled on the prognostic values of the American Joint Committee for Cancer (AJCC) 8th Edition Cancer Staging System [21]. Patient characteristics were age, menopausal status, family history, breast region, tumor quadrant, histopathological subgroups, stage, axillary stage, ER status, progesterone receptor (PR) status, human epidermal growth factor (HER) 2 status, Ki67, perineural invasion (PNI), lymphovascular invasion (LVI), tumor grade, mitotic index, molecular subgroup (Luminal A, Luminal B, triple-negative breast cancer [TNBC] and HER2-enriched) [22], extensive intraductal component (EIC), breast surgery type, axillary surgery type, whether chemotherapy was received, chemotherapy type, whether Trastuzumab was received, duration of tamoxifen (TAM) use, and duration of aromatase inhibitor (AI) use.

### Treatment decision

Adjuvant systemic treatment decisions were made based on risk factors such as tumor size, tumor grade, nodal involvement, and age of patients, as defined in institutional guidelines. Adjuvant RT was applied to the whole breast/chest wall and regional lymph nodes as 50 Gy / 2 Gy in 25 fractions (fr) for 5 weeks in breast cancer patients with lymph node involvement, and a dose of 10–12 Gy / 2 Gy in 5–6 fr, was applied to the tumor bed. In patients without lymph node involvement, 50 Gy / 2 Gy in 25 fr was applied to the whole breast area for 5 weeks, and 10–12 Gy / 2 Gy in 5–6 fr was applied to the tumor bed. Partial breast irradiation was not administered to any analyzed patient. In boost treatments, external RT doses were applied as electron or photon therapy. In order to shorten the treatment period only during the COVID-19 pandemic, BED3 equivalent was calculated and 45 Gy / 2.5 Gy in 18 fr was applied to the whole breast/chest wall and supraclavicular/axillar region, and 10 Gy / 2 Gy in 5 fr to the tumor bed to patients with lymph node involvement. In patients without lymph node involvement, 45 Gy / 2.5 Gy in 18 fr was applied to the whole breast and 10 Gy / 2 Gy in 5 fr was applied to the tumor bed.

### Immunohistochemical procedure and evaluation

ER and PR positivity assessments were made using Primary Novocastra monoclonal antibodies. ER and PR positivity is determined as ≥ 1% of tumor cell nuclei being immunoreactive [23]. Immunohistochemical **(**IHC) analyses were performed following DAKO Herceptest scoring. Complete solid staining of the cell membrane in more than 10% of the tumor cells was interpreted as HER2 positivity and was scored 3+. FISH was used to confirm HER2 positivity in weak to moderate cell membrane staining in more than 10% of the tumor cells and scored 2+. Faint, incomplete cell membrane staining in more than 10% of the tumor cells was scored 1 + and interpreted as trace negative. No staining was interpreted as HER2-negative and scored 0 [24, 25]. The Ki67 score was defined as the percentage of stained tumor cell nuclei and was analyzed in paraffin sections using MIB-1 IHC staining. The stained section was examined using a standard light microscope with a 40x objective and 10 × 10 graticule. At least 1000 stained tumor cell nuclei in ten high-power fields (× 40) were considered evaluable [26]. In the St. Gallen International Consensus Panel in 2011, four main subtypes have been approved in the classification scheme (22). According to the presence or absence of ER, PR, and HER2, these molecular subtypes have been defined as Luminal A (ER and PR-positive, HER2-negative, low Ki67), Luminal B (ER and/or PR positive, HER2-positive or high Ki67), HER2-Enriched (ER and PR-negative, HER2-positive) and Triple-Negative (TNBC) (ER, PR, HER2-negative).

Patients’ medical records were used for follow-up data. The follow-up of our patients who completed the adjuvant treatment process was done every 3 months for the first 2 years, every 6 months from the third to the fifth year, and once a year after the 5th year. While creating SPSS data, locoregional recurrence and/or distant metastasis dates, date of death, or last control date were recorded to calculate DFS and OS.

### Statistical analysis

Propensity score matching was performed between the groups that did and did not receive RT using an optimal matching algorithm (optimum, 1:1) applying age, stage, axillary stage, and LVI variables [27]. Variables significantly different between the two groups or considered clinically important by Pearson’s chi-square test were used to create propensity scores. DFS and OS after PSM were calculated using the Kaplan-Meier method. Numerical results are expressed as the mean ± standard deviation and categorical results are shown as n (%). Survival curves were generated using the Kaplan–Meier method, and the significance of survival differences among the selected variables was compared using the log-rank test [28]. Univariate Cox regression analysis was used to estimate hazard ratios. Then, multivariate Cox regression analysis with a backward elimination method was used to estimate hazard ratios and to identify independent prognostic factors [29]. All reported *p* values are two-sided, and *p* values below 0.05 were considered significant. Data analysis was performed using SPSS version 20.0 (IBM SPSS Statistics for Windows, Version 20.0. Armonk, NY: IBM Corp.).

## Results

In our analysis to measure the effectiveness of RT, we excluded 799 of 2811 breast cancer patients with stage IIIB-IV, followed by 243 patients with ductal carcinoma in situ. Out of 1769 of our remaining invasive breast cancer patients, 1580 received RT and 189 did not. Characteristics of patients with invasive breast cancer who underwent RT and those who did not are shown in Table 1. In order to measure the effect of RT properly, propensity score analysis was performed. Balancing of both groups was done according to age, stage, nodal involvement, and LVI (Table 2).

**Table 1:** Distribution table of clinical, histopathological and treatment features before and after propensity score analysis, except for the clinical and histopathological features used for propensity score analysis of the patient groups with and without RT.

	Pre-Propensity Score			Post-Propensity Score		
	Without RT *n* = 189 (%)	With RT *n* = 1580 (%)	*p*	Without RT *n* = 189 (%)	With RT *n* = 189 (%)	*p*
**Menopausal Status**						
Premenopausal	65 (34.40)	672 (42.50)	.124	65 (34.4)	68 (36.0)	.746
Postmenopausal	124 (65.60)	908 (57.50)		124 (65.6)	121 (64.0)	
**Sex**						
Female	187 (98,90)	1570 (99,40)	.362	187 (98.90)	187 (98.90)	1.000
Male	2 (1.10)	10 (0.60)		2 (1.10)	2 (1.10)	
**Family History**						
Positive	58 (30.70)	510 (32.30)	.828	58 (30.70)	73 (38.60)	.105
Negative	131 (69.30)	1070 (67.70)				
**Breast Site**						
Right	91 (48.10)	796 (50.40)		91 (48.10)	100 (52.90)	.429
Left	95 (50.30)	765 (48.40)	.544	95 (50.30)	88 (46.60)	
Bilateral	3 (1.60)	19 (1.20)		3 (1.60)	1 (0.50)	
**Tumor Quadrant**						
Lateral	115 (60.80)	1005 (63.60)		115 (60.80)	118 (62.40)	.381
Medial	46 (24.30)	329 (20.80)	.202	46 (24.30)	34 (18.00)	
Areola	21 (11.10)	179 (11.30)		21 (11.10)	25 (13.20)	
Multifocal	7 (3.80)	67 (4.24)		7 (3.80)	11 (6.40)	
**Histopathological Subgroup**						
IDC	147 (77.70)	1268 (80.30)	.377	147 (77.70)	147 (77.70)	.440
ILC	16 (8.50)	135 (7.80)		16 (8.50)	17 (9.00)	
Others	26 (13.80)	177 (11.20)		26 (13.80)	25 (13.30)	
**ER Status**						
Positive	158 (83.60)	1319 (83.50)	.220	158 (83.60)	163 (86.20)	.472
Negative	31 (16.40)	261 (16.50)		31 (16.40)	26 (13.80)	
**PR Status**						
Positive	133 (70.40)	1106 (70.00)	.308	133 (70.40)	143 (75.60)	.247
Negative	56 (29.60)	474 (30.00)		56 (29.60)	46 (24.40)	
**HER2 Status**						
Positive	37 (19.60)	363 (23.00)	.474	37 (19.60)	21 (11.10)	.022
Negative	152 (80.40)	1217 (77.00)		152 (80.40)	168 (88.90)	
**Ki67**						
< 15	75 (39.70)	552 (35.00)	.108	75 (39.70)	72 (38.10)	.752
≥ 15	114 (60.30)	1028 (65.00)		114 (60.30)	117 (61.90)	
**PNI**						
Present	30 (15.90)	288 (18.20)	.518	30 (15.90)	27 (14.30)	.666
None	159 (84.10)	1292 (81.80)		159 (84.10)	162 (85.70)	
**Tumor Grade**				140 (74.10)	140 (74.10)	.762
I	40 (21.20)	251 (15.90)	.015			
II	97 (51.30)	800 (50.60)				
III	52 (27.50)	529 (33.50)				
**Mitotic Index**						
I	56 (29.60)	348 (22.10)	.09	56 (29.60)	63 (33.30)	.732
II	96 (50.80)	844 (53.40)		96 (50.80)	92 (48.70)	
III	37 (19.60)	388 (24.50)		37 (19.60)	34 (18.00)	
**Subgroup**						
Luminal A	57 (30.20)	460 (29.10)		57 (30.20)	64 (33.90)	.177
Luminal B	104 (55.00)	891 (56.40)	.626	104 (55.00)	103 (54.50)	
Triple Negative	21 (11.10)	203 (12.80)		21 (11.10)	20 (10.60)	
HER2-Enriched	7 (3.70)	26 (1.70)		7 (3.70)	2 (1.00)	
**EIC**						
Positive	4 (2.10)	250 (15.80)	< .001	4 (2.10)	9 (4.80)	.158
Negative	185 (97.90)	1330 (84.20)		185 (97.90)	180 (95.20)	
**Breast Surgery Type**						
BCS	28 (14.80)	939 (59.40)	< .001	28 (14.80)	147 (77.70)	< .001
MRM	161 (85.20)	641 (40.60)		161 (85.20)	42 (22.30)	
**Axillary Surgery Type**						
SLND	80 (42.30)	416 (26.30)	< .001	80 (42.30)	101 (53.40)	.201
AC	109 (57.70)	1164 (73.70)		109 (57.70)	88 (46.60)	
**Status of Receiving Chemotherapy**						
None	35 (18.50)	160 (10.10)		35 (18.50)	39 (20.60)	.861
Neoadjuvant	10 (5.30)	196 (12.40)	< .001	10 (5.30)	9 (4.80)	
Adjuvant	144 (76.20)	1224 (77.50)		144 (76.20)	141 (74.60)	
**Type of Chemotherapy**						
AC + TXT	91 (48.10)	878 (55.70)		91 (48.10)	85 (45.00)	.192
FAC-FEC-TAC + TXT	52 (27.50)	674 (42.50)	< .001	52 (27.50)	62 (32.80)	
RIBO + PALBO + PERTUZUMAB + CMF	8 (4.20)	28 (1.80)		8 (4.20)	2 (1.10)	
**Trastuzumab Status**						
Received	24 (12.70)	290 (18.40)	.075	24 (12.70)	15 (7.90)	.176
Did not receive	165 (87.30)	1290 (81.60)		165 (87.30)	174 (92.10)	
**Duration of TAM Use**						
≤ 5 years	79 (41.80)	564 (35.70)	.577	79 (41.80)	70 (37.00)	.303
>5 years	7 (3.70)	104 (6.60)		7 (3.70)	13 (6.90)	
**Duration of AI Use**						
≤ 5 years	89 (47.10)	751 (47.50)	.366	89 (47.10)	92 (48.70)	.707
>5 years	25 (13.20)	220 (13.90)		25 (13.20)	28 (14.80)	

RT: Radiotherapy, ER: Estrogen Receptor, PR: Progesterone Receptor, HER2: Human Epidermal Growth Factor Receptor 2, PNI: Perineural Invasion, EIC: Extensive Intraductal Carcinoma, BCS: Breast Conserving Surgery, MRM: Modified Radical Mastectomy, SLND: Sentinel Lymph Node Dissection, AC: Axillary Curettage, AC: Adriamycin, Cyclophosphamide, TXT: Taxotere, FAC: Cyclophosphamid, Adriamycin, 5-Fulourouracil, FEC: 5-Fulouracil, Epirubicine, Cyclophosphamide, TAC: Taxotere, Adriamycin, Cyclophosphamid, RIBO + PALBO: Ribociclib + Palbociclib, CMF: Cyclophosphamide, Methotrexate, Fluorouracil, TAM: Tamoxifen, AI: Aromatase Inhibitor

**Table 2:** Distribution of clinical and histopathological features of propensity score analysis, used to balance invasive breast patients who did not receive radiotherapy with the patient group who received RT, before and after analysis

		Without RT (*n* = 189)	With RT (*n* = 1580)	*p*	Without RT (*n* = 189)	With RT (*n* = 189)	*p*
Age	< 35	9 (4.80)	82 (5.20)	0.059	9 (4.80)	9 (4.80)	1.000
	36–50	62 (32.80)	654 (41.40)		62 (32.80)	62 (32.80)	
	> 50	118 (62.40)	844 (53.40)		118 (62.40)	118 (62.40)	
Stage	I	86 (45.50)	383 (24.20)	< 0.001	86 (45.50)	86 (45.50)	1.000
	IIA	68 (36.00)	506 (32.00)		68 (36.00)	68 (36.00)	
	IIB	17 (9.00)	317 (20.10)		17 (9.00)	17 (9.00)	
	IIIA	18 (9.50)	374 (23.70)		18 (9.50)	18 (9.50)	
Axillary Stage	N0	153 (81.00)	745 (47.20)	< 0.001	153 (81.00)	153 (81.00)	1.000
	1–3	19 (10.10)	508 (32.20)		19 (10.10)	19 (10.10)	
	4–9	17 (9.00)	327 (20.70)		17 (9.00)	17 (9.00)	
LVI	No	49 (25.90)	738 (46.70)	< 0.001	49 (25.90)	49 (25.90)	1.000
	Yes	140 (74.10)	842 (53.30)		140 (74.10)	140 (74.10)	

LVI: Lymphovascular Invasion

**Fig. 1:**
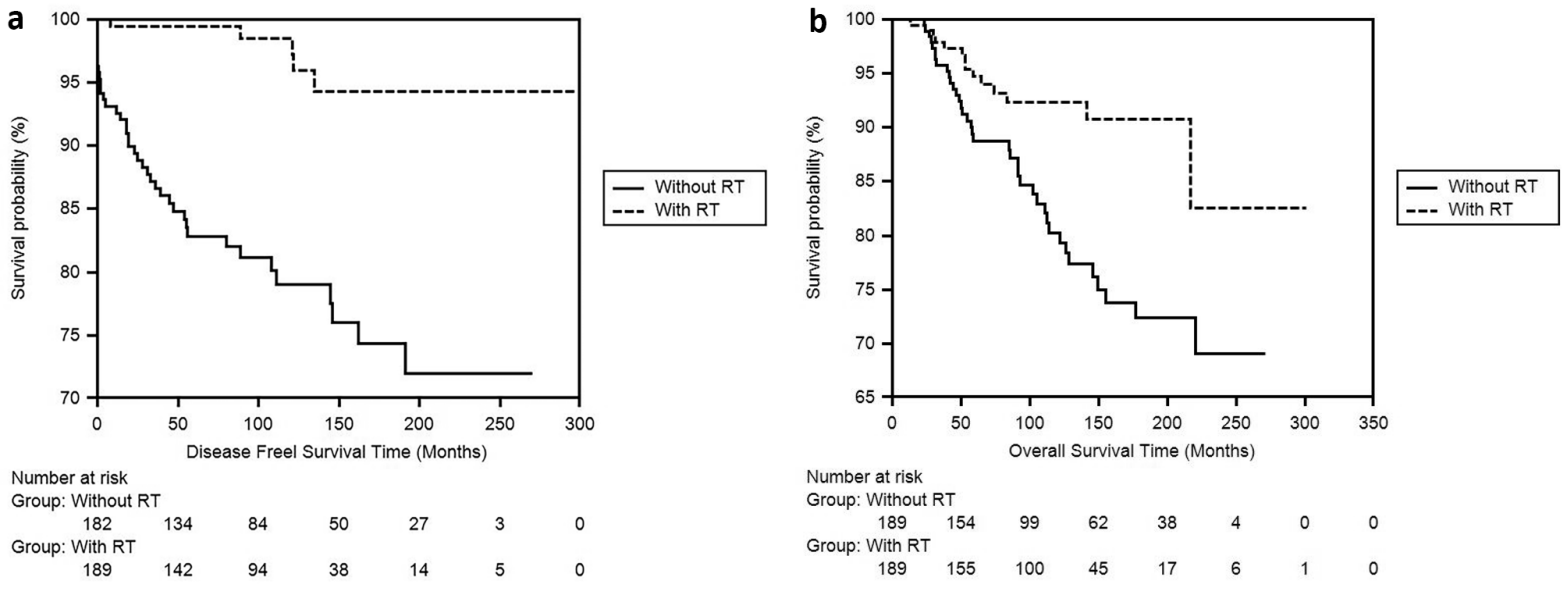
**a** Comparison of DFS with radiotherapy treated breast cancer patients in which 189 breast cancer patients who did not receive radiotherapy were balanced with the propensity score by Kaplan-Meier. **b** Comparison of OS with radiotherapy treated breast cancer patients in which 189 breast cancer patients who did not receive radiotherapy were balanced with the propensity score by Kaplan-Meier

First, DFS and OS times were compared between the patients who received and did not receive radiotherapy. In the RT and non-RT groups, DFS was 257.42 ± 5.46 (246.72- 268.13) and 208,96 ± 8,15 (192,97–224,94) months respectively (***p = < 0.001)***, and OS was 272,46 ± 8,68 (255,43–289,49) and 219,05 ± 7,32 (204,70–233,41) months respectively (*p* = .002). Both DFS and OS times were significantly longer in patients who received RT (Fig. 1a and b).

**Fig. 2:**
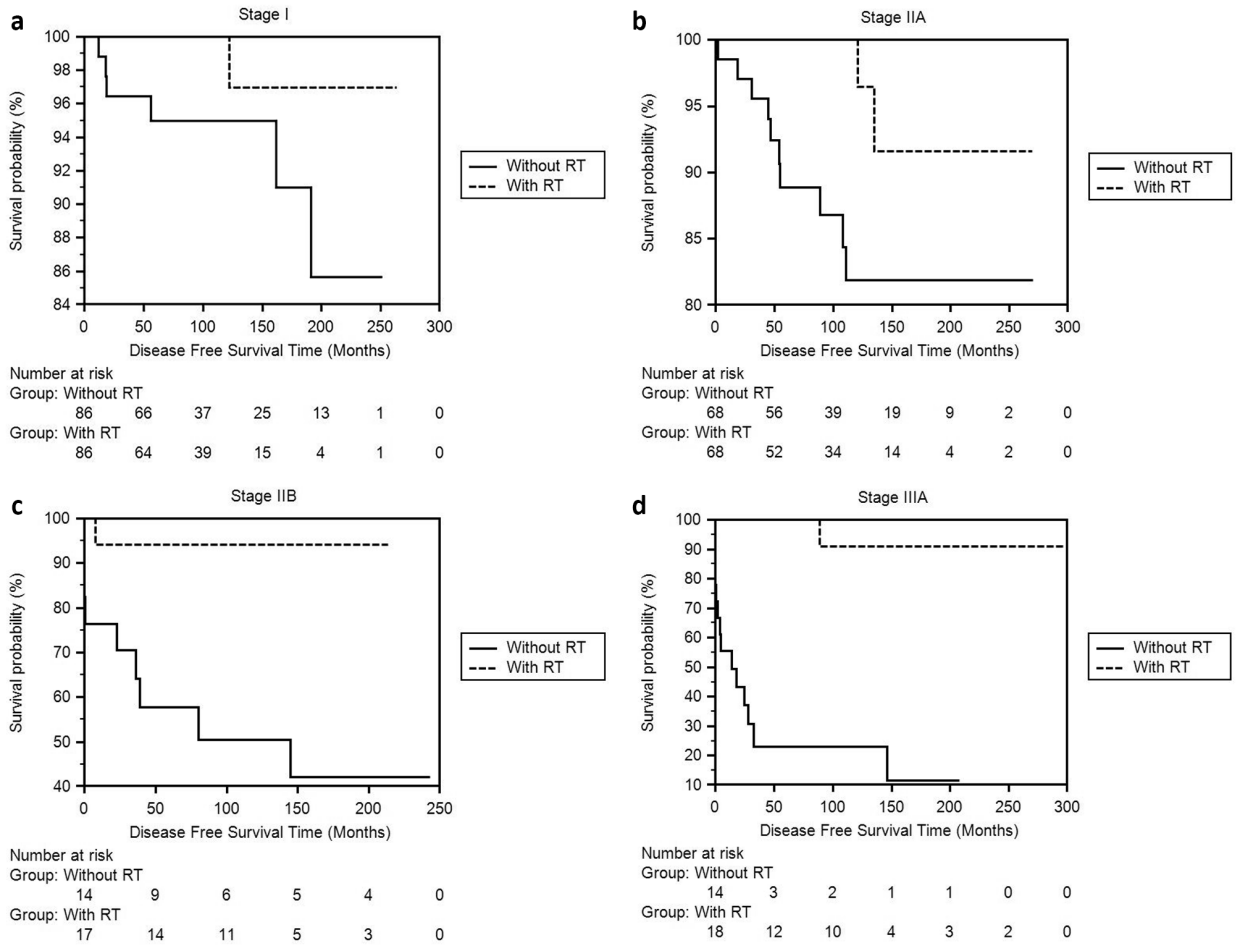
**a, b, c, d.** Comparison of DFS times of Stage I, IIA, IIB, IIIA invasive breast cancer patients who did not receive RT with the patient group who received RT using the Kaplan-Meier method after propensity score balancing

**Fig. 3:**
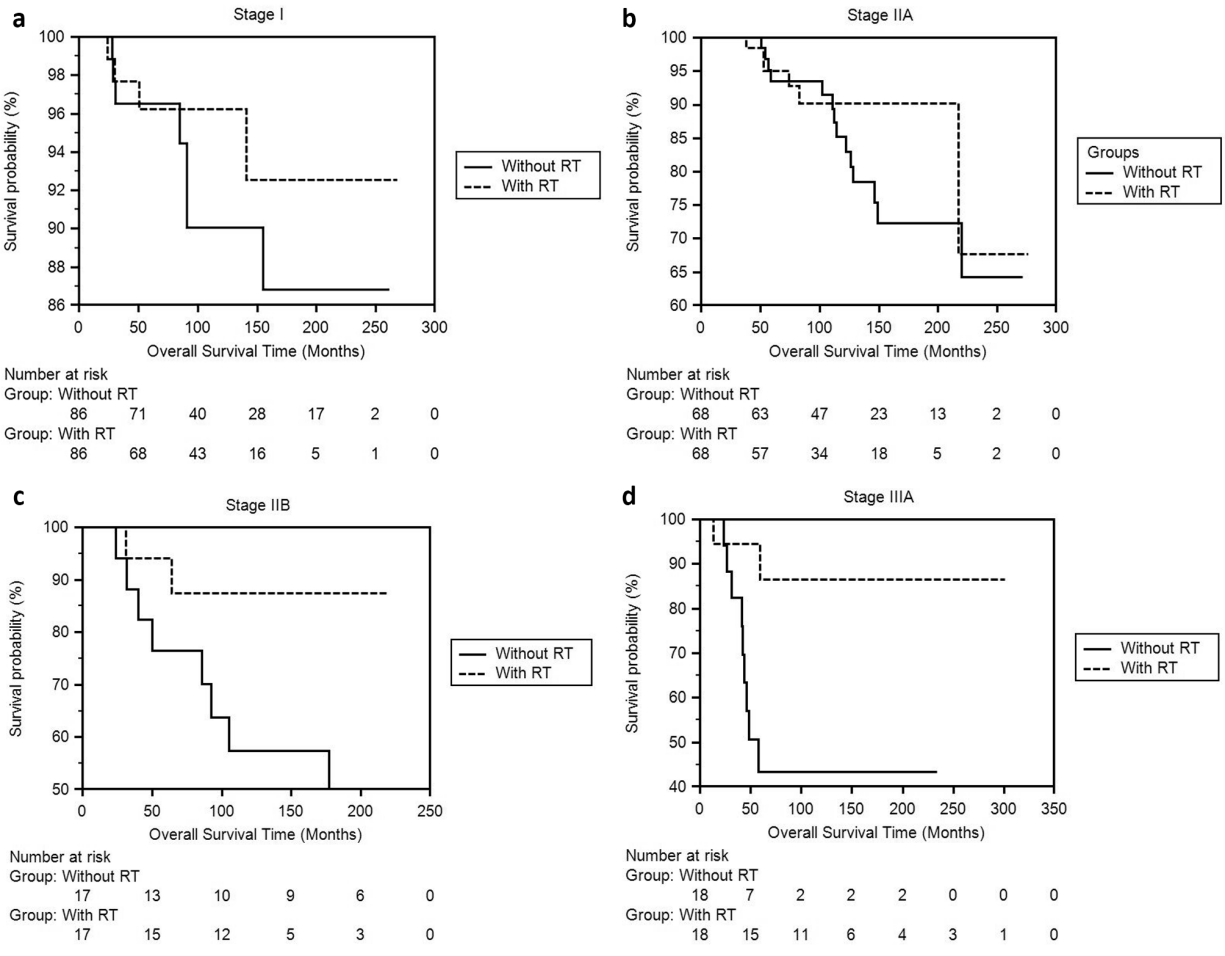
**a, b, c, d.** Comparison of OS times of Stage I, IIA, IIB, IIIA invasive breast cancer patients who did not receive RT with the patient group who received RT using the Kaplan-Meier method after propensity score balancing

The second step calculated DFS and OS times according to the stages (Table 3). In the patient group that received RT, DFS time was significantly longer in Stages IIA, IIB, and IIIA, other than Stage I (Fig. 2a, b, c and d). Although survival times were longer in the RT group in all stages, statistical significance was detected in stages IIB and IIIA (Fig. 3a, b and c 3d).

**Table 3:** Comparison of DFS and OS times calculated by the Kaplan-Meier method with the RT patient group after balancing with propensity score analysis of 189 invasive breast cancer patients in our series who did not receive RT.

**DFS**	**Without RT (months)**	**With RT (months)**	*p*
Stage I	233.08 ± 6.96 ( 219.42-246.74 )	258.72 ± 4.20 ( 250.48-266.97 )	0.108
Stage IIA	232.44 ± 10.87 ( 211.12-253.75 )	257.25 ± 8.01 ( 241.54-272.95 )	0.029
Stage IIB	126.51 ± 26.77 ( 74.04-178.98 )	200.94 ± 11.69 ( 178.01-223.87 )	***0.005***
Stage IIIA	49.43 ± 18.50 ( 13.16–85.70 )	277.18 ± 17.94 ( 242.01-312.34 )	***< 0.001***
**OS**	**Without RT (months)**	**With RT (months)**	***p***
Stage I	238,42 ± 8,13 (222,48–254,37 )	254,57 ± 6,68 (241,46–267,68 )	*0.223*
Stage IIA	222,05 ± 11,12 ( 200,25–243,85 )	241,91 ± 14,21 ( 214,05-273,82 )	***0.225***
Stage IIB	161,76 ± 22,10 ( 118,43–205,09 )	198,43 ± 14,29 ( 170,41–226,45 )	***0.065***

**Fig. 4:**
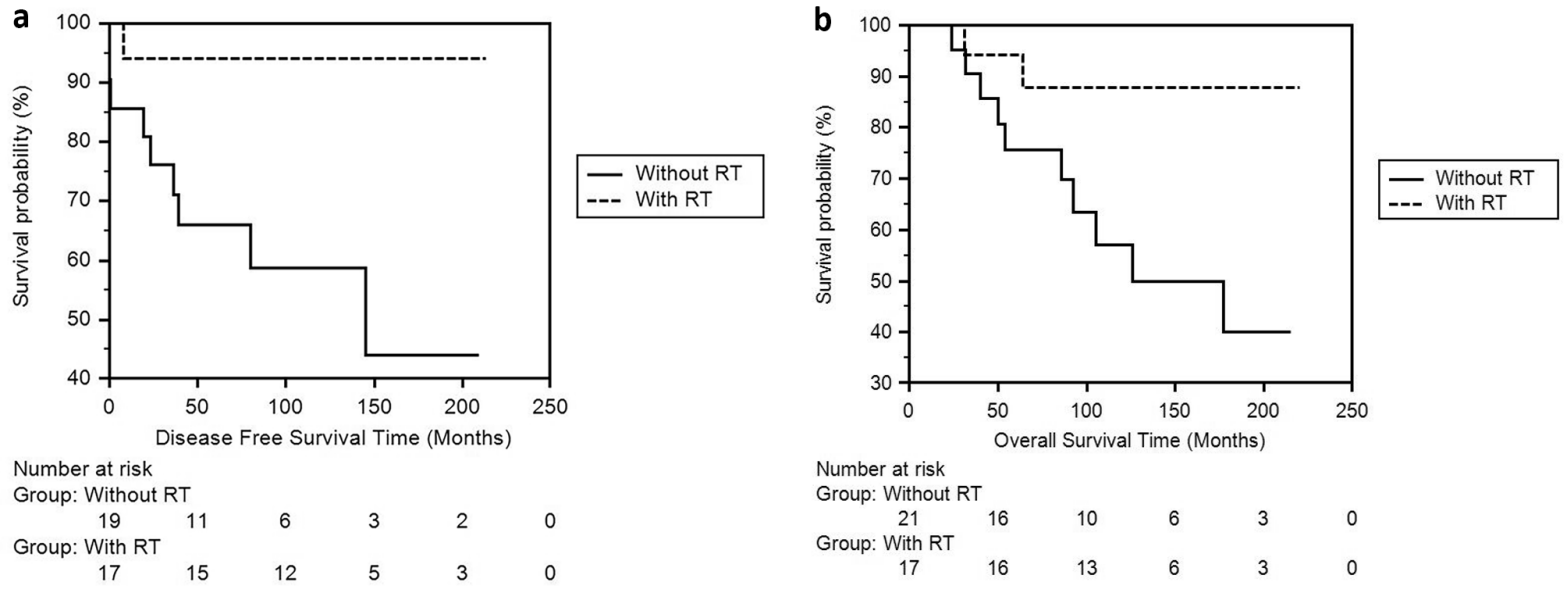
**a** Comparison of DFS times between the groups that received RT and those that did not receive RT in our series with invasive breast cancer patients with 1–3 positive lymph nodes using the Kaplan-Meier method. **b** Comparison of OS times between the groups that received RT and those that did not receive RT in our series with invasive breast cancer patients with 1–3 positive lymph nodes using the Kaplan-Meier method

In the third step, we evaluated our 19 patients in N1 stage with 1–3 lymph node involvement, where the use RT is controversial. We compared the 19 N1 patient groups who received RT with the 19 patients who did not receive RT and calculated the DFS times was 202,21 ± 10,50 (181,62–222,79) and 148,82 ± 24,91 (99,99–197,65) months respectively (*p* = .011) and OS times was 200,85 ± 12,79 (175,77–225,92) and 166,90 ± 20,39 (126,93–206,82) months respectively (*p* = .055). We found that both DFS and OS times were longer in the RT group, with statistical significance and very close to significance (Fig. 4a and b).

**Fig. 5:**
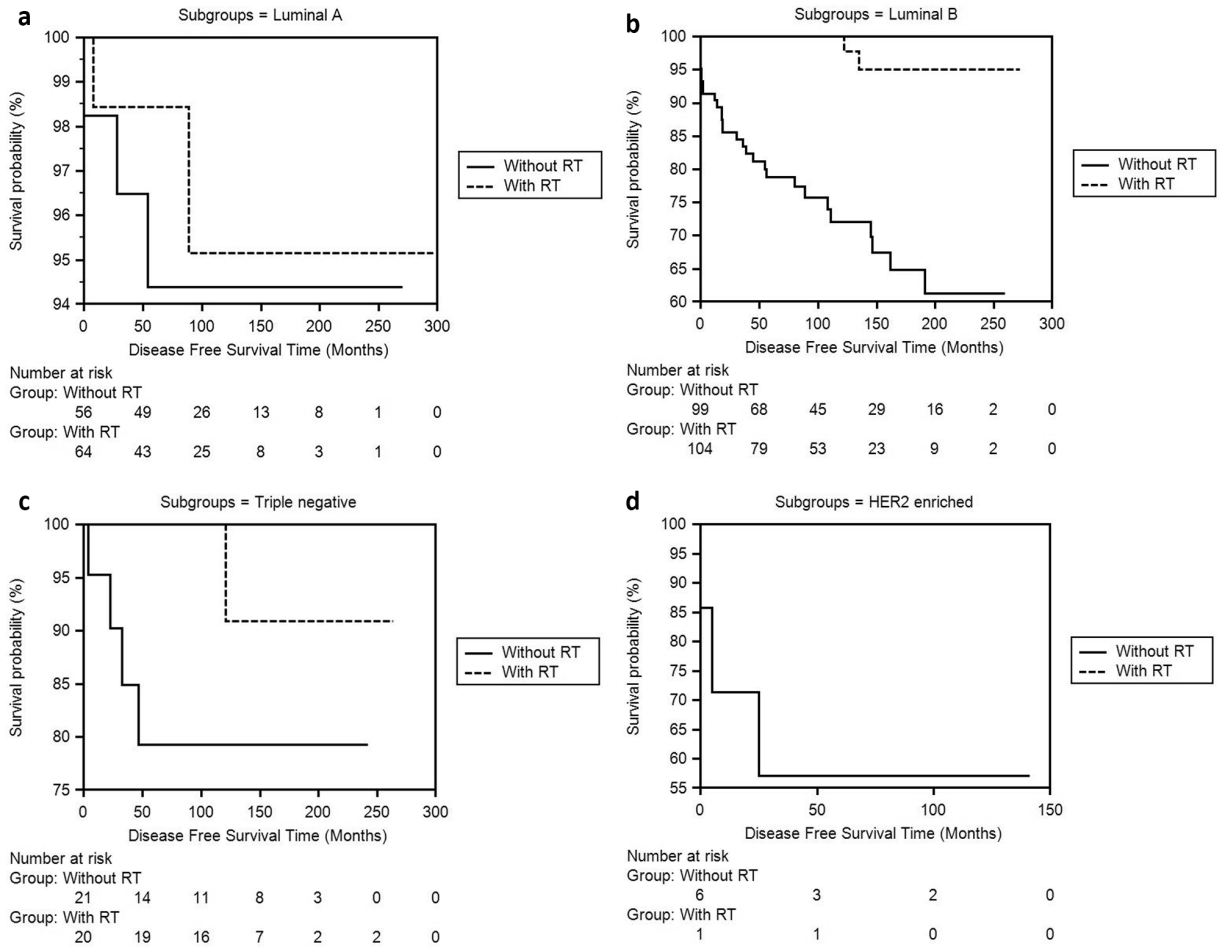
**a, b, c, d**. Comparison of DFS times between the RT and non-RT groups of the Luminal A, Luminal B, Triple Negative, HER2-Enriched subgroup of 189 invasive breast cancer patients in our series using the Kaplan-Meier method

**Fig. 6:**
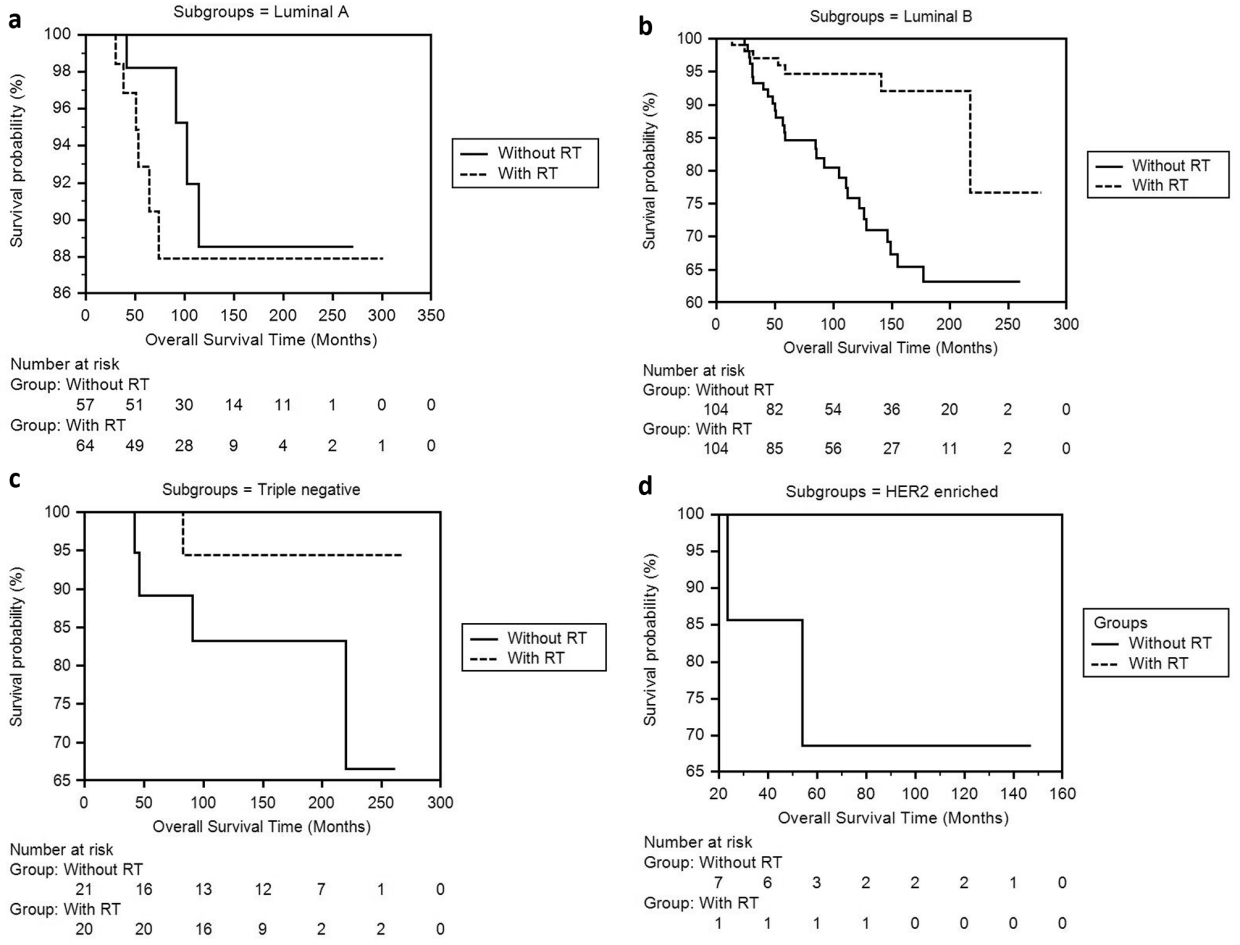
**a, b, c, d.** Comparison of OS times between the RT and non-RT groups of the Luminal A, Luminal B, Triple Negative, HER2-Enriched subgroup of 189 invasive breast cancer patients in our series using the Kaplan-Meier method

In step four, we analyzed the two patient groups that received and did not receive RT according to their molecular subgroups. We examined DFS and OS times in both groups according to Luminal A, Luminal B, TNBC, and HER2-enriched subgroups. In the Luminal B subgroup, the DFS duration in the groups receiving RT and not receiving RT was 264.83 ± 4.95 (255.13-274.54) and 187.09 ± 11.06 (165.41-208.78) months (*p* < .001), and OS times were 252.29 ± 10.54 (231.62-272.97) and 197.74 ± 9.72 (178.69–216.80) months (*p* = .001) respectively (Table 4; Figs. 5a, b, c and d and 6a, b, c and d).

**Table 4:** Comparison of DFS and OS times of patients who received RT and those who did not receive RT according to subgroups after propensity score analysis of invasive breast cancer patients

	DFS (months)			OS (months)		
	**Without RT** **months ± sd** **95% CI** **(Lower-Upper Bound)**	**With RT** **months ± sd** **95% CI** **(Lower-Upper Bound)**	*p*	**Without RT** **months ± sd** **95% CI** **(Lower-Upper Bound)**	**With RT** **months ± sd** **95% CI** **(Lower-Upper Bound)**	*p*
Luminal A	256.48 ± 7.59 (241.59-271.37)	284.70 ± 7.97 (269.07-300.33)	.620	250.64 ± 9.69 (231.64-269.63)	271.13 ± 11.59 (248.41-293.85)	.482
Luminal B	187.09 ± 11.06 (165.41-208.78)	264.83 ± 4.95 (255.13-274.54)	***< .001***	197.74 ± 9.72 (178.69–216.80)	252.29 ± 10.54 (231.62-272.97)	***.001***
Triple Negative	197.55 ± 19.87 (158.61–236.50)	250.09 ± 12.30 (225.96-274.21)	.145	220.57 ± 18.13 (185.02-256.12)	257.72 ± 9.98 (238.14-277.29)	.201
HER2-Enriched	54.85 ± .17 (5.00-141.03)	79 ± .13 (0.00-162.23)	.475	75.91 ± 0.13 (23.40–147.00)	85 ± 0.27 (22.10-112.30)	.558

In addition, we examined the effect of RT on the whole group, stage, number of involved lymph nodes and subgroups in our patients with invasive breast cancer, as well as DFS and OS times using Cox regression univariate and multivariate analysis tests (Table 5). In our series, when 189 patients who did not receive RT were balanced with 189 patients who received RT, it was determined that RT reduced the risk of recurrence and metastasis in the entire group by 8 times (*p* < .001) in univariate analysis and 12.8 times in multivariate analysis (*p* < .001). RT also reduced the risk of death by 2.5 times (*p* = .003) in univariate analysis and 2.8 times (*p* = .001) in multivariate analysis. When the risk analysis was evaluated according to stages, although RT was not statistically significant in stage I, it reduced the risk of recurrence and metastasis by 4.9 times (*p* = .145) and the risk of death by 1.7 times (*p* = .422). In stage IIA, RT significantly reduced the risk of recurrence and metastasis by 4.7 times (*p* = .047) in univariate analysis, and 2 times (*p* = .141) in multivariate analysis. It also reduced the risk of death by 1.8 times (*p* = .232) in univariate analysis and 2.3 times (*p* = .028) in multivariate analysis for stage IIA. In stage IIB, RT reduced the risk of recurrence and metastasis by 10.6 times (*p* = .025) in univariate analysis and 9.7 times (*p* = .02) in multivariate analysis. Risk of death was reduced by 3.9 times (*p* = .086) in univariate analysis, and 5.4 times (*p* = .034) in multivariate analysis for patients in stage IIB who received RT. In stage IIIA, RT reduced the risk of recurrence and metastasis by 26.3 times (*p* = .002) in univariate analysis and 77 times (*p* = .957) in multivariate analysis. Risk of death was reduced by 6 times (*p* = .002) in univariate analysis and 125 times (*p* = .955) in multivariate analysis for patients in stage IIIA who received RT. When the risk analysis was made according to N stage, it was found that RT reduced the risk of recurrence and metastasis by 5.2 times (*p* = .149) and risk of death by 1.7 times in stage N0. For stage N1, risk of recurrence and metastasis was reduced 9.3 times (*p* = .036) in univariate analysis and 1.5 times (*p* = .662) in multivariate analysis in RT recipients. RT also reduced the risk of death by 4.1 times (*p* = .076) in univariate analysis and 1.4 times (*p* = .662) in multivariate analysis for patients in N1 stage. When the risk analysis was made in accordance to molecular subgroups, it was found that RT reduces risk of recurrence and metastasis by 15.2 times (*p* < .001) and risk of death by 3.8 times (*p* = .002) in Luminal B subgroup.

**Table 5:** Effect of RT on DFS and OS in patients with invasive breast cancer and risk factors through Cox regression univariate and multivariate analysis

Cox Regression	Univariate				Multivariate			
	*p*	HR	95% CI		*p*	HR	95% CI	
			Lower	Upper			Lower	Upper
**DFS**								
**RT Effect on the Whole Group**	***< .001***	**.125**	**.049**	**.317**	***< .001***	**.078**	**.030**	**.205**
**Stage I**	*.145*	.205	**.024**	**1.728**	**1**	**Reference**		
**Stage IIA**	***.047***	.215	**.047**	**.983**	*.141*	2.032	.790	5.226
**Stage IIB**	***.025***	.094	**.012**	**.743**	***.020***	**9.733**	**1.421**	**66.657**
**Stage IIIA**	***.002***	**.038**	**.005**	**.295**	*.957*	.013	.000	6787
**N0**	***.009***	**.193**	**.056**	**.660**	**1**	**Reference**		
**N1**	***.036***	**.108**	**.013**	**.864**	*.662*	.662	.104	4.201
**N2**	***.001***	**.035**	**.004**	**.269**	*.924*	2112	.000	1809
**Subgroups**								
**Luminal B**	***< .001***	**.066**	**.016**	.276	**1**	**Reference**		
**Triple Negative**	*.183*	.226	.025	2.023	*.177*	.487	.171	1.383
**Luminal A**	*.623*	.638	.106	3.829	***.004***	**.245**	**.093**	**.645**
**HER2-Enriched**	*.650*	.039	.00	30.69	*.851*	1.130	.315	4.059
**OS**								
**RT Effect on the Whole Group**	***.003***	**.394**	**.213**	**.729**	***.001***	**2.892**	**1.542**	**5.426**
**Stage I**	*.422*	.604	**.176**	**2.070**	**1**	**Reference**		
**Stage IIA**	*.232*	.556	.213	1.455	***.028***	**2.333**	**1.093**	**4.979**
**Stage IIB**	*.086*	.256	.054	1.214	***.034***	**5.408**	**1.133**	**25.818**
**Stage IIIA**	***.022***	**.166**	**.036**	**.766**	*.955*	.008	.000	7.207
**N0**	***.149***	**.572**	**.268**	**1.222**	**1**	**Reference**		
**N1**	***.076***	**.244**	**.052**	**1.159**	*.662*	.728	.176	3.020
**N2**	***.020***	**.161**	**.034**	**.752**	*.935*	1087.8	.000	9999
**Subgroups**								
**Luminal B**	***.002***	**.265**	**.116**	**.608**	**1**	**Reference**		
**Triple Negative**	*.233*	.261	.029	2.378	.069	.391	.142	1.074
**Luminal A**	*.486*	1.568	.442	5.565	***.023***	**.435**	**.212**	**.889**
**HER2-Enriched**	*.710*	.039	.00	68.60	.868	.881	.198	3.919

## Discussion

Thanks to studies proving that RT increases long-term survival rates in breast cancer as a result of reducing locoregional recurrence and systemic metastasis rates, it has been understood that the spectrum hypothesis is the hypothesis that most accurately describes breast cancer to date [7, 8, 13, 30–32]. Moreover, the contribution of RT was confirmed to be of similar magnitude not only for patients with four or more lymph node-positive invasive breast cancer, but also for breast cancer patients with one to three positive axillary lymph node infiltrations [6, 7, 31–33]. In this situation, thanks to these very important results, breast cancer patients with 1–3 lymph node involvement will be guaranteed to receive RT today, where late side effects are minimized thanks to modern techniques. Therefore, we analyzed the DFS and OS times of patients in our series who did not receive RT for various reasons.

Since our series had a strict and long follow-up period, we performed statistical analysis by balancing it with the propensity score in order to measure the effect of RT objectively but as it deserves. In our series, the DFS was significantly longer in the patient group receiving RT in all stages except stage I (*p* = .108, *p* = .029, *p* = .005, *p* < .001). Although the OS was longer in the patient group receiving RT in all stages, we found statistical significance in stage IIIA and close to statistical significance in stage IIB. However, when we measured the effect of RT in our series on patients with invasive breast cancer with 1–3 lymph node involvement, both DFS (*p* = .011) and OS (*p* = .055) times were longer in the RT group. It may be possible to interpret this situation as the intertwining of the spectrum hypothesis and the abscopal effect of RT. In this way, it may be possible to explain the contribution of lymph node irradiation to DFS and OS in patients with lymph node involvement as it is known to prevent the migration of tumor cells to distant organs and elicit an antitumor immune response through the abscopal effect of radiation [34, 35]. Jatoi et al. [35, 36] reported that the abscobal effect of RT application in breast cancer is in line with the breast cancer spectrum hypothesis in terms of integrity, cause and effect. Additionally, Fortin A. et al. [37] proved in 1999 that breast cancer patients with local failure showed worse survival compared to patients with local control. In conclusion, we aimed to emphasize that removing RT from the treatment protocol of a breast cancer patient requires serious evaluation and justification.

Randomized studies showed that combining surgery with RT not only reduces the risk of locoregional recurrence, but also substantionally lowers the risk of metastasis and death [6–8, 13, 38]. The key question has been addressed by two major randomized studies investigating whether RT after breast-conserving surgery for early stage breast cancers should be performed locally or locoregionally [39, 40]. These studies have shown that locoregional RT is not only protective against locoregional recurrence, but also reduces the risk of metastasis. Although no benefits in survival in the 10-year follow-up results of locoregional RT were observed, it is suggested that it may provide a survival advantage over a longer follow-up period.

The 2020 National Comprehensive Cancer Network (NCCN) guidelines recommend postoperative RT for patients with 4 positive axillary lymph nodes after total mastectomy, and for those with 1–3 positive axillary lymph nodes [41]. In our series, among patients with N0 invasive breast cancer who received RT, risk of recurrence and metastasis decreased by 5.2 times, and the death risk decreased by 1.7 times. In N1 patients, the recurrence and metastasis risk decreased by 9.3 times, and the death risk decreased by 4.1 times. In N2 patients, the recurrence and metastasis risk decreased by 28.6 times, and the death risk decreased by 6.2 times. The 2019 St. Gallen International Consensus Guidelines recommend RT for N1 patients with TNBC subgroup [42]. In another recent study among N1 invasive breast cancer patients, post-mastectomy RT not only contributed to DFS and OS, but also showed better locoregional outcomes in both Luminal A, Luminal B and TNBC subgroups. However, information regarding subgroups and RT application varies considerably in the literature [43].

Whelan et al. [39] reported that patients with ER-negative or PR-negative tumors benefited more from regional nodal irradiation compared to patients with ER-positive or PR-positive tumors. However, He et al. [44] stated that the TNBC subgroup is more radioresistant compared to Luminal subgroups. Two other studies evaluating the effect of RT also found that the HER2-enriched subgroup is more radioresistant than Luminal and TNBC subgroups [45, 46]. Kyndi et al. [46] found that in patients who received RT after mastectomy, the local recurrence rate was higher in TNBC compared to luminal subgroups.

Due to contradictory results in the literature, we analyzed the effect of RT in our series according to subgroups. We found that patients with Luminal B invasive breast cancer benefited significantly more from RT compared to other subgroups. However, in all subgroups, both DFS and OS durations were longer in the group that received RT. Risk of recurrence, metastasis and death was significantly lower only in Luminal B subgroup in Cox regression univariate analysis. In multivariate Cox regression analysis, when the Luminal B subgroup was taken as a reference, it reduced the recurrence/metastasis risk by 4.1 times (*p* = .004) and the death risk by 2.3 times (*p* = .023) compared to the Luminal A subgroup. We understand that there is not complete clarity in the literature on this matter, but as more series investigate and share their findings with the literature, clearer information may become available.

## Conclusion

Use of RT in breast cancer treatment maintains its high effectiveness as a result of the complementary nature of its abscopal effect and the spectrum theory. Considering that RT, which has less toxicity thanks to modern treatment approaches, is used considering the risks and benefits, we aimed to highlight that the possible apiscopal effects of radiotherapy should not be overlooked to achieve the longest survival. Just as systemic treatments have become widely accepted for significantly reducing the risk of breast cancer recurrence and death during the first three to five years after diagnosis, we highlight the importance of considering the contribution of delayed systemic effects of RT due to the abscopal effect in reducing the risk of recurrence, metastasis, and death. We also recognize that the molecular subgroup of the tumor may also be taken into consideration in future practices when using RT.

### Electronic supplementary material

Below is the link to the electronic supplementary material.

Supplementary Material 1

## Data Availability

https://datadryad.org/stash/share/MIpMdhlFyzPUFJG45cq_IbC0FEaEJvDVroO64lfmhZ0.

## Abbreviations

DFS: Disease-Free Survival
OS: Overall Survival
PSM: Propensity Score Matching
RT: Radiotherapy
PMRT: Post-Mastectomy Radiotherapy
NICE: National Institute for Health and Care Excellence
AJCC: American Joint Committee for Cancer
ER: Estrogen Receptor
PR: Progesterone Receptor
HER2: Human Epidermal Growth Factor-2 status
PNI: Perineural Invasion
LVI: L ymphovascular Invasion
TNBC: Triple-Negative Breast Cancer
EIC: Extensive Intraductal Component
TAM: Tamoxifen
AI: Aromatase Inhibitor
IHC: Immunohistochemical
NCCN: National Comprehensive Cancer Network
monoclonal MIB1: Monoclonal Antibody
DAKO: Ki-67 IHC MIB-1 pharmDx (Dako Omnis) contains optimized reagents and the protocol required to complete an IHC staining procedure of FFPE specimens using the Dako Omnis
