# Supplementary material for: Retrospective evaluation of the contribution of radiotherapy to survival in breast cancer treatment with propensity score based on stage and subgroup
Source: Radiat Oncol. 2024 Jun 26;19:83. doi: 10.1186/s13014-024-02474-x (PMC11210162; doi:10.1186/s13014-024-02474-x)
Supplement: Supplementary file 1 — Supplementary Material 1 [file 13014_2024_2474_MOESM1_ESM.pdf]

T.C.
TRAKYA ÜNİVERSİTESİ REKTÖRLÜĞÜ
Tıp Fakültesi Dekanlığı
Girişimsel Olmayan Bilimsel Araştırmalar Etik Kurulu

Sayı : E-22121724-050.04.04-454876 24.05.2023
Konu : Kararlar

Sayın Prof. Dr. Ruşen COŞAR

Trakya Üniversitesi Tıp Fakültesi Bilimsel Araştırmalar Etik Kurulu'nun 08.05.2023 tarihli toplantısında almış olduğu, yürütücüsü olduğunuz; "Meme Kanserinden Sağkalımda Radyoterapinin Katkısının Propensity Skoru Aracılığıyla İncelenmesi: Tüm Evreler Ama Özellikle Evre IIA" adlı TÜTF-GOBAEK 2023/195 protokol no.lu çalışmanıza ait 05 no.lu gündem görüşülmüş olup, imza aşaması tamamlandıktan sonra elden teslim edilecektir.
Bilgilerinizi rica ederim.

Dr. Öğr. Üyesi Fatma Gülsüm ÖNAL
Bilimsel Araştırmalar Etik Kurulu Başkanı

Ek:Elden Teslim Edilecektir

Bu belge, güvenli elektronik imza ile imzalanmıştır.

Belge Doğrulama Kodu :BSD8804ZRH Pin Kodu :09372 Belge Takip Adresi : https://www.turkiye.gov.tr/trakya-universitesi-ebys

Adres : Trakya Üniversitesi Rektörlüğü Tıp Fakültesi Dekanlığı Balkan Yerleşkesi 22030 Edirne
Telefon : 2842357653 Faks : 2842357652
e-Posta:dekanlik@trakya.edu.tr Web:http://tipfak.trakya.edu.tr/
Kep Adresi : trakyauni@hs01.kep.tr
Bilgi için : Yeşim ERDOĞAN
Unvanı : Sekreter
